# Microbiota Involved in the Degradation of *Tremella fuciformis* Polysaccharide and Microbial Enzymatic Potential Revealed by Microbiome and Metagenome

**DOI:** 10.3390/microorganisms13020263

**Published:** 2025-01-25

**Authors:** Xiao Song, Guangning Chen, Long Zheng, Jingjing Shen, Changhu Xue, Yaoguang Chang

**Affiliations:** College of Food Science and Engineering, Ocean University of China, 1299 Sansha Road, Qingdao 266404, China; songxiao9694@126.com (X.S.);

**Keywords:** *Tremella fuciformis*, polysaccharide, microbiome, metagenome, glycoside hydrolases

## Abstract

*Tremella fuciformis*, as a traditional edible fungus in Asian countries, is rich in polysaccharides with a variety of bioactivities. Nevertheless, its high molecular weight and complex structure have caused limitations in its application and structural analysis. In this study, we successfully screened a *Tremella fuciformis* polysaccharide-degrading bacterium from the soil by enriching and screening. The mixed bacterium consisted mainly of *Verrucomicrobium* (55.4%) and *Lysobacter* (43.8%), which released extracellular enzymes that enabled the degradation of *Tremella fuciformis* polysaccharides. The functional annotation using microbiome and metagenome combined with bioinformatics revealed its active carbohydrate metabolism, binding, and catalysis. It exposed the enzymatic potential of the bacterium and provided a basis for the exploration of hydrolytic enzymes for hardly degradable polysaccharides in fungi.

## 1. Introduction

*Tremella fuciformis* Berk. is the fruiting body of the fungus in the *Tremella* of the Ascomycota with desirable taste, high nutritional value, and large-scale cultivation, which has a great potential in the development of prepared foods, natural food additives, and meal replacement [1,2]. It is a class of edible fungi traditionally in Asian countries, growing in mild and humid environments, and is mostly distributed and cultivated in the deep forests and high mountains in China [3]. *Tremella fuciformis* (TF) is rich in active ingredients such as polysaccharides, proteins, and dietary fiber with low calorie and fat content. It has a variety of biological activities such as enhancing immunity [4], reducing blood sugar and blood lipids [5], antioxidant and anti-aging effects [6,7], etc., which are popular among people as well as attract the attention of researchers. *Tremella fuciformis* polysaccharides (TFPs) could improve gut health by stimulating the growth of Bacteroides, *Phascolarctobacterium* and *Lachnoclostridium*, as well as promoting the production of SCFA [8]. There were studies showing that the low-molecular-weight TFPs have stronger antioxidant and immunomodulatory activities than those with high molecular weight [9]. TFPs are not only an attractive health food but also a promising structural component for improving gel properties [10] and delaying starch gelatinization [11].

The polysaccharides in TF are dominated by acidic heteropolysaccharides. Most of the previous reports indicated that TFPs consist of a linear (1→3) linked α-d-mannose backbone and contain residues or side chains of β-d-xylose, β-d-glucuronic acid, and β-d-fucose [2,12,13]. Nevertheless, the structure resolution of TFPs is still unclear due to the high heterogeneity, complex side chains, and the lack of corresponding enzymes. Meanwhile, its application is also limited by high molecular weight and high viscosity, resulting in poor selectivity for the preparation of active oligosaccharides.

Soil is recognized as the most diversified microbial habitat on Earth in terms of species diversity and community magnitude [14]. Complex carbohydrates in the form of structural and storage polysaccharides constitute the largest reservoir of metabolizable carbon in the biosphere. It is a ubiquitous source of energy that provides fuel for microbial elements in terrestrial ecosystems. Carbohydrate utilization is inextricably linked to the continued presence of microorganisms in the soil [15]. Soil microorganisms have been a major source for the separation of novel biocatalysts [16], among which the degradation of organic matter by glycoside hydrolases (GHs) is one of the important functions of soil [17]. Carbon flows into the soil from plant material, providing microorganisms with the opportunity to metabolize carbon compounds and synthesize genes that encode carbohydrate enzymes [18]. The abundance of Bacteroidetes, Verrucomicrobia and Proteobacteria in the soil is closely related to carbohydrate metabolism. In particular, they provide a natural multi-enzyme synergistic environment for the degradation of complex polysaccharides in the wild.

Metagenomics is an effective method for the direct sequencing of DNA obtained from environmental samples, which circumvents the dependence on single bacterial cultures. In recent years, it has often been applied to explore and discover potential enzymes produced by microorganisms in natural environments, especially soil, water, and animal gut [19]. Amsale Melkamu et al. [20] utilized a shotgun metagenomics approach to assemble, predict, annotate, and identify carbohydrate hydrolases for the first time from the microbiome of a forest soil. Pang et al. [21] cloned and characterized three different families of endoglucanases from the metagenomes of compost soils. Chantorn et al. [22] expressed highly active mannanase and xylanase from lignocellulolytic bacteria *Paenibacillus polymyxa* BTK01 and *Bacillus subtilis* BTK07 isolated from organic rice field soils. Metagenome combined with bioinformatics analysis has great potential for application in the discovery of novel potential glycoside hydrolases.

In this study, we enriched and screened TF-degrading bacteria from soils in several locations, and successfully obtained a mixed bacteria from soil. The extracellular enzymes released by them could realize the degradation of TFPs. The microbiome was used to explore the species in this bacterial group that can utilize *Tremella fuciformis* polysaccharides, and metagenome was used in combination with bioinformatics to reveal the enzymatic potential.

## 2. Materials and Methods

### 2.1. Materials

**Figure 1:**
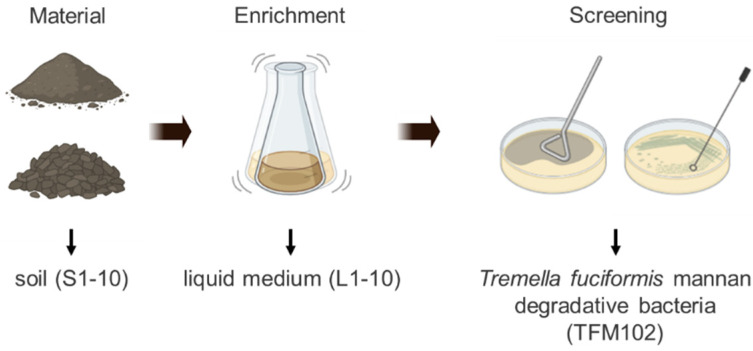
Screening process and naming of microbial samples.

*Tremella fuciformis* was purchased from the market. The soil samples were collected from the origins of *Tremella fuciformis* and alpine environments with ecological differences. The cultivation substrates of *Tremella* were purchased from Linyi Shandong, Ningde Fujian, and Guangzhou Guangdong. The soils were collected from Lao Mountain and Xiaozhu Mountain in Qingdao, Tai Mountain in Taian, and Jiuzhaigou in Sichuan. The samples’ naming is shown in Figure 1.

### 2.2. Extraction of Tremella fuciformis Polysaccharides

Crude polysaccharides were extracted from *Tremella fuciformis* powder with water at 80 °C for 6 h, insoluble *Tremella* residue was removed by centrifugation at 5000 rpm for 20 min, and then the supernatant was mixed with 3 times the volume of ethanol (95% *v*/*v*) overnight and centrifuged at 5000 rpm for 20 min to recover the polysaccharide precipitate. The precipitate was dissolved again, and the starch and protein were removed with amylase (0.1%) and papain (0.1%), followed by dialysis and freeze-drying to obtain the TFPs.

### 2.3. Bacterial Screening and Purification

Each sample was enriched and induced using liquid medium with a sole carbon source, that is, 0.2 g of the sample was added to 5 mL of TFP medium (2 mg/mL TFP with 10% inorganic salt). The culture was shaken at 30 °C for 72 h at 160 rpm. Enrichment and induction were repeated 3 times at 5% inoculum. The polysaccharide utilization was eluted by high-performance size exclusion chromatography and detected by a refractive index detector (HPSEC-RID) (Agilent 1260, Agilent Technologies, Santa Cruz, CA, USA), which eluted with a TSKgel SuperAW4000 column (Cytiva, Uppsala, Sweden). The eluent was 0.2 M NaCl, and the flow rate was set at 0.4 mL/min. The bacteria that were able to utilize the substrate were further screened by coating in TFP solid medium (TFP liquid medium supplemented with 2% agar) and cultured at 30 °C for 72 h. Single colonies were picked and inoculated in liquid medium and incubated at 30 °C and 160 rpm for 48–72 h. The culture supernatant and cell lysate supernatant were taken to treat the substrate, and the enzyme activity inside and outside the cell was determined using HPSEC-RID.

### 2.4. 16S rDNA Amplicon Sequencing

Amplicon sequencing was performed on 7 samples, including S10, L2, L6, L7, L8, L10, and TFM102. The TIANamp soil DNA kit (Tiangen Biotech, Beijing, China) was used for DNA extraction of the soil sample (S10) and cytrimethylammonium bromide (CTAB) for other samples. The genomic DNA of the samples was extracted and then the purity and concentration of the DNA were detected by an agarose gel electrophoresis. Then, 16S rRNA genes of distinct regions (16SV4) were amplified using a specific primer (GTGCCAGCMGCCGCGGTAA, GGACTACHVGGGTWTCTAAT) with the barcode. All PCR reactions were carried out with Phusion^®^ High-Fidelity PCR Master Mix (New England Biolabs, Ipswich, MA, USA). The constructed library was quantified by Qubit and Q-PCR. After the library was qualified, it was sequenced by illumina NovaSeq 6000 (Illumina, San Diego, CA, USA). Raw data were obtained, first spliced, and filtered to obtain clean data. Amplicon Sequence Variant (ASV) clustering and species classification analysis were then performed based on valid data by QIIME 2 (version 2022.2) [23,24]. Species annotations were made for the representative sequences of each ASV by database Silva 138.1 (≥70% identity), and the corresponding species information and species-based abundance distribution were obtained at the phylum to genus level. Representative sequences of the top 100 genus were obtained by multiple-sequence comparison in order to further investigate the phylogenetic relationships of species at the genus level. At the same time, ASVs were analyzed for abundance, and Alpha diversity calculations, Venn plots, and petal plots were conducted to obtain species richness and uniformity information within the sample. Alpha diversity was calculated from 3 indices in QIIME 2, including Chao1, Shannon, and Simpson.

### 2.5. Metagenome Sequencing and Functional Annotation

TFM102 was inoculated in TFP liquid medium at 5% inoculum. After passaging three times, the bacteria were collected for DNA extraction. Metagenome sequencing based on Pacbio platforms was entrusted to gene sequencing companies. The sequencing was performed using HiFi-reads, and the sequencing depth was about 31×. The whole genome of the dominant species was obtained by HiFi Metagenome assembly. GTDB (Genome Taxonomy Database, version 1.3.0, http://gtdb.ecogenomic.org/) was used to classify the assembled sequences at the genomic level [25]. GO [26] (Gene Ontology, http://geneontology.org/, accessed on 18 March 2024), KEGG [27] (Kyoto Encyclopedia of Genes and Genomes, http://www.genome.jp/kegg/, accessed on 18 March 2024), CAZY [28] (Carbohydrate-Active enZYmes Database), and SwissProt [29] (http://www.ebi.ac.uk/uniprot/, accessed on 18 March 2024) were used for gene function annotation.

### 2.6. Determination of Macro-Transcriptomics and Differential Expression Analysis

TFM102 was inoculated in TFP and Glu medium (2 mg/mL glucose with 10% inorganic salt) at 5% inoculum and passed to the fourth generation. Bacteria at logarithmic growth period (18 h) were collected for RNA extraction. Macro-transcriptomics by mRNA-seq technology were entrusted to gene sequencing companies. Software such as RSEM [30] was used to compare the mRNA sequences measured to the TFM102 metagenome. DESeq2 [31] or edgeR [32] was used for differential expression analysis. Genes with|log2(Fold Change)| > 1 & *p*adj < 0.05 were assigned as differentially expressed.

## 3. Results and Discussion

### 3.1. Enrichment of Microbiota from Different Sources

**Figure 2:**
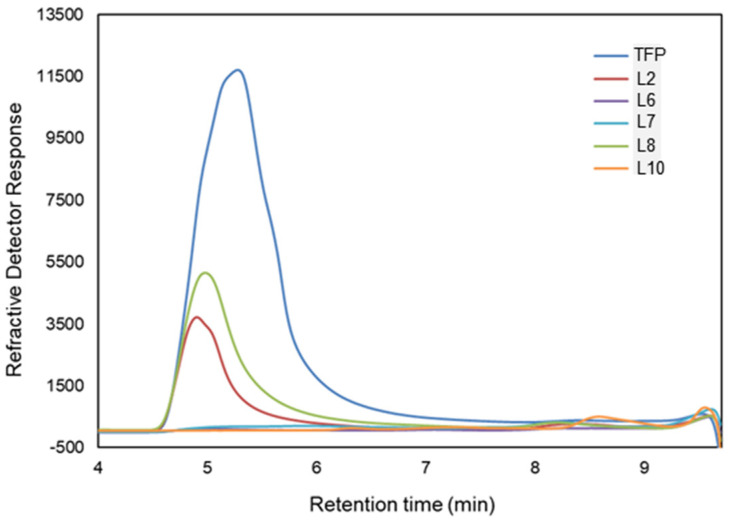
HPSEC-RID chromatogram of changes in *Tremella fuciformis* polysaccharides (TFPs) content of the culture medium after utilization by enriched microorganisms from different sources. The TSKgel SuperAW4000 column was utilized for the determination of polysaccharides. The peak located at retention time 4.6–6.0 min was TFPs.

TF is grown and cultured in various areas of China, including wild- and basswood-cultured TF in Sichuan, as well as bag-cultured TF in Fujian, Guangzhou, Linyi, and others. In order to screen for bacteria with TFP degradation ability, 10 samples of microbial environments were collected from sources in different regions (see Table S1 for sample source information). TFPs were used as the sole carbon source to enrich and induce the bacteria in the samples. The content and molecular weight changes of carbon sources in the culture solution were determined using HPSEC as a measure of the carbon source utilization by the microbiota. The results showed that microorganisms in samples L2, L6, L7, L8, and L10 were able to degrade and substantially utilize TFPs (Figure 2). The degradation rates of L6, L7, and L10 all reached about 99%, while L2 and L8 could reach 85% and 67%, respectively. Meanwhile, microorganisms involved in TFPs degradation and utilization were greatly enriched and exhibited advantages.

Aiming to investigate the correlation between the bacteria from different sources which could utilize TFPs, the enriched bacterial fluids L2, L6, L7, L8, and L10 were analyzed by amplicon analyses, including their species diversity, relative species abundance, and functional predictions.

**Figure 3:**
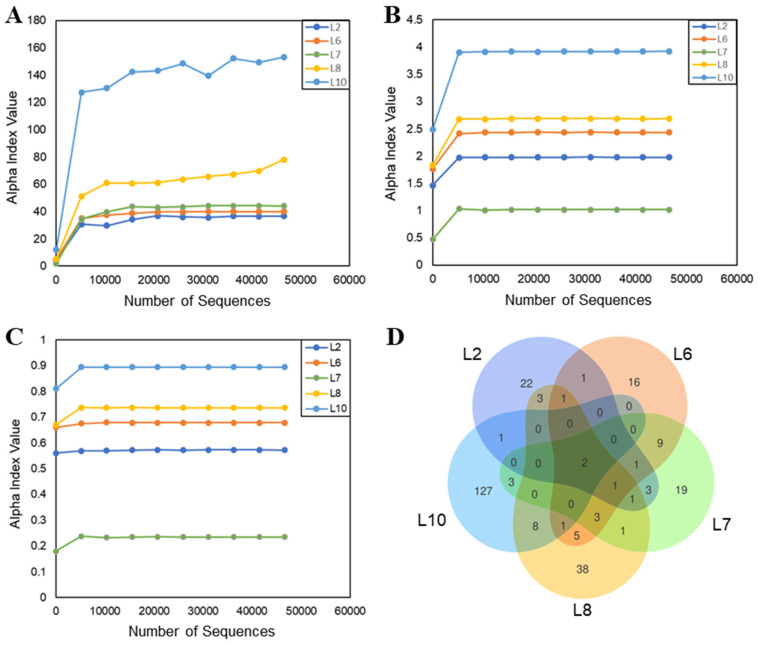
Chao1 (**A**), Shannon (**B**), and Simpson (**C**) dilution curves and ASV Venn diagrams (**D**) of five TFP-degrading bacteria. The numbers in the overlapping part of the circles in the Venn represent the feature ASVs common among the samples, and the numbers in the non-overlapping part represent the feature ASVs unique to the samples.

Alpha diversity analysis (Chao1, Shannon, and Simpson indices) showed that L10 had high species diversity and prominent dominant species, followed by L8 (Figure 3A–C). The Venn diagram showed the common, unique feature ASVs between different samples, and each circle in the diagram represented a sample. The numbers in the overlapping part of the circles represent the feature ASVs common among the samples, and the numbers in the non-overlapping part represent the feature ASVs unique to the samples. As can be seen by the Venn, species overlap was low between the five samples, with only two common ASVs (Figure 3D). L10 had the most unique ASVs, followed by L8. All of these results were positively correlated with the excellent growth capacity and stability of L10 and L8, especially L10. Moreover, L10 and L8 had eight common ASVs, suggesting that the two more dominant TFP-degrading bacteria have partially identical characteristic species.

**Figure 4:**
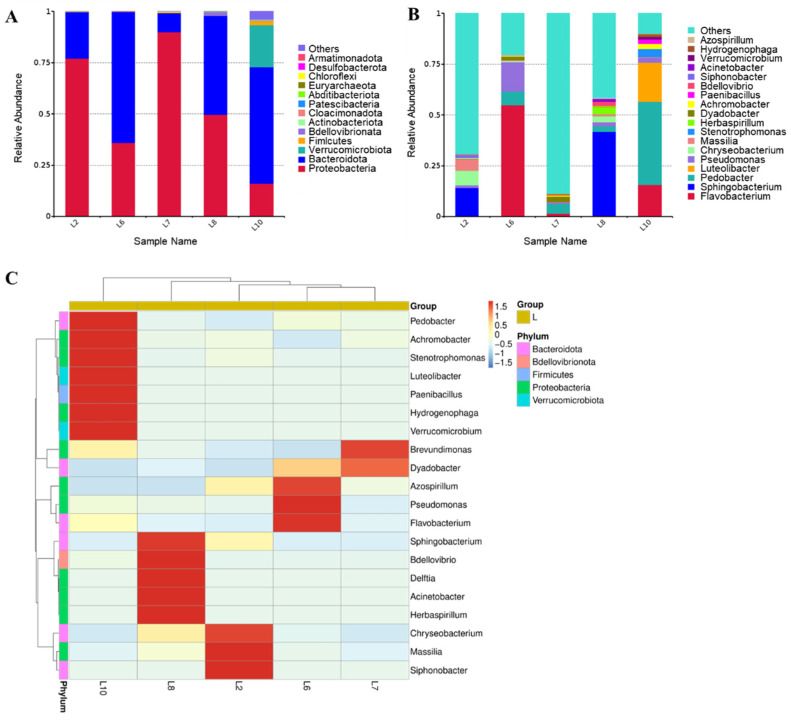
Histograms of species relative abundance at the phylum (**A**) and genus (**B**) levels, and a clustered heat map of species abundance at the genus level (**C**). “Others” indicates the sum of the relative abundances of all phyla other than the 10 in the figure. The horizontal coordinate of the heat map is the sample name, and the vertical coordinate is the annotation information of the species. The clustering tree on the left side of the figure is the species clustering tree. The color gradient from blue to red indicates the relative abundance of each species within the samples, where blue represents low abundance and red represents high abundance.

As can be seen from the relative abundance histograms of phylum (Figure 4A), L2, L6, L7, and L8 all consisted mainly of Proteobacteria and Bacteroides. Verrucomicrobia was only represented in L10. At the genus level, 24, 18, 22, 43, and 52 genera were annotated in L2, L6, L7, L8, and L10, respectively, and the top 18 genera of the five samples and their proportions are shown in Figure 4B. *Sphingobacterium* was the dominant genus in L2 and L8, while *flavobacterium* and *pedobacter* occupied a higher proportion in L6 and L10, respectively, and most species in L7 were not annotated to genus. As shown in the clustering heat map (Figure 4C), there was no significant correlation between the five samples. It demonstrates that the species structure of the five bacterial groups was completely different. The dominant species in the bacterial groups may have been produced due to the induction of the TFP medium. Microbiota functions of the five samples were predicted with a focus on the annotation of glycoside hydrolases. The glycoside hydrolases involved in the five bacterial groups were summarized by EC annotations (EC: 3.2.1.), among which up to 55 glycoside hydrolase species were included in L10, much more than the other samples.

In summary of the results, L10 simultaneously possesses strong utilization capacity, excellent diversity, and obvious dominant species, as well as being involved in more abundant carbohydrate metabolism functions. It laid the foundation for its stable carbon source utilization and extracellular enzyme activities in subsequent studies.

### 3.2. Enzymatic Activity and Bacterial Structure Analysis of Bacteria Capable of Utilizing TFP

**Figure 5:**
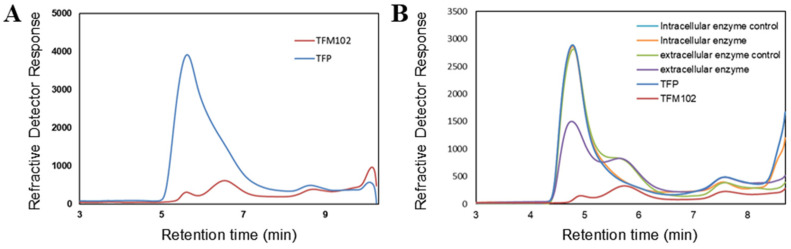
HPSEC-RID chromatogram of changes in TFPs content of the culture medium after utilization by TFM102 (**A**) and its intracellular and extracellular enzymes (**B**). The TSKgel SuperAW4000 column was utilized for the determination of polysaccharides.

In order to further enrich and screen the dominant bacteria, L10 was cultured in a solid medium. The colonies which grew well were picked for carbon source utilization assay to verify, and finally, a mixed bacterium was obtained, which could not be further purified. It could utilize TFPs stably up to more than 90% (Figure 5A), and was named as TFM102. Unfortunately, the target bacteria in the remaining four samples could not be colonized on a solid medium.

The enzyme activities in the supernatant of TFM102 culture fluid (extracellular enzyme activity) and the supernatant of the broken bacterium (intracellular enzyme activity) were determined using HPSEC (Figure 5B). The results show that the extracellular enzyme of TFM102 could reduce the peak of TFPs up to 48.2%, indicating that it performed degradation activity. The polysaccharide-degrading enzymes released by TFM102 may be mainly extracellular. Relative to the substrate control and the culture medium control, the TFPs peak was not delayed after extracellular enzyme degradation but showed a substantial decrease directly, proving a strong activity. Meanwhile, only a few oligosaccharides were produced, which could be attributed to the presence of other extracellular enzymes that converted the oligosaccharides into smaller products. The results confirm that TFM102 produces TFP-degrading enzymes.

**Figure 6:**
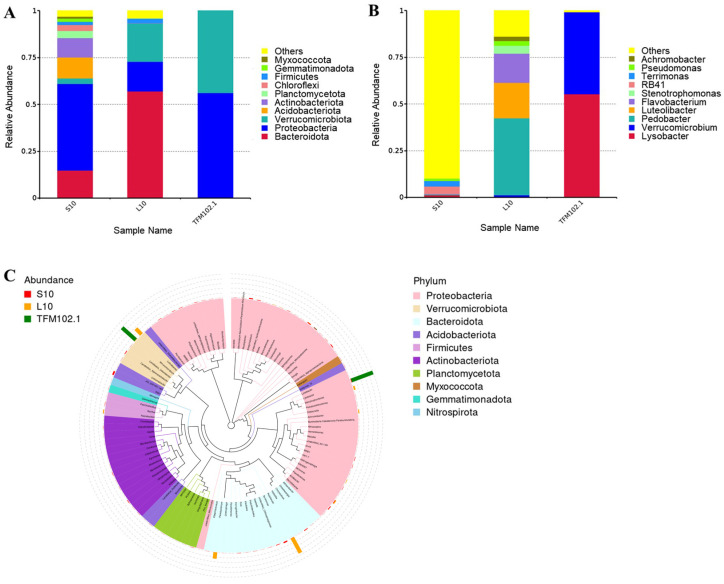
Relative abundance histograms of species at the phylum (**A**) and genus (**B**) level, and evolutionary tree of species at the genus level (**C**). “Others” indicates the sum of the relative abundances of all phyla other than the 10 in the figure. The phylogenetic tree was constructed using representative sequences at the genus level. The colors of the branches and wedges denote their corresponding phyla. The stacked bar chart positioned outside the radial tree illustrates the abundance distribution of the respective genera across different samples.

To characterize the changes in the microbial structure of TFM102 from soil to enrichment and screening, the species diversity and relative abundance of TFM102 were compared with its soil sample S10 and enrichment sample L10 (Figure 6A, B). In the process of enriching microorganisms in the soil, at the level of phylum, species of Bacteroidetes, Proteobacteria, and Verrucomicrobiota entrenched their predominance. Acidobacteriota and Actinobacteriota, which occupied a certain percentage of the soil, disappeared after enrichment. Bacteria of Acidobacteriota play an important role in the ecosystem [33], while the most bacteria in Actinobacteriota are saprophytes, both of which are ubiquitous soil bacteria. At the genus level, 246, 52, and 3 genera were annotated in S10, L10, and TFM102, respectively, and the top 10 genera of the three samples and their proportions were shown in Figure 6B. There were no obvious dominant species in S10, consistent with the diverse and complex microbial environment in the soil [20]. After enrichment, obvious dominant species appeared in L10, including *Pedobacter*, *Flavobacterium* of Bacteroidetes, and *Luteolibacter* of Verrucomicrobiota, suggesting that the liquid medium containing TFPs influenced the changes in microbial structure.

TFM102 consisted of two dominant bacteria in similar proportions, with *Verrucomicrobium* and *Lysobacter* accounting for 43.8% and 55.4%, respectively. Based on the carbon source utilization of TFM102, it could be demonstrated that *Verrucomicrobium* and *Lysobacter* were fully involved in the degradation of TFPs and utilized them. Species of Verrucomicrobia are widely distributed in soil, water bodies, and animal feces. Several of these species, including *Verrucomicrobium*, have been shown to be associated with the degradation of polysaccharides [34,35,36]. The genome of *Verrucomicrobium* sp. strain GAS474 isolated by Pold et al. contains several families of carbohydrate-active enzymes, especially glycoside hydrolases [37]. A strain from Verrucomicrobia isolated from paddy soil by Chin et al. had the ability to degrade pectin and cellulose [35]. *Lysobacter* is a new biological control agent known for its diverse fungal lytic enzymes. It is capable of producing a variety of extracellular enzymes such as chitinase, β-1,3-glucanase, cellulase, etc., which play essential roles in inhibiting the germination of fungal spores and lysing mycelium [38]. Both Verrucomicrobiota and Proteobacteria are classical microbial branches producing carbohydrate-active enzymes with the potential to degrade complex polysaccharides. Unexpectedly, the composition of dominant species in TFM102 was completely different from that of L10. The growth of species in L10 may be more dependent on nutrients in the original soil. Nevertheless, the species evolutionary tree (Figure 6C) shows that *Pedobacter*, *Luteolibacter* and *Flavobacterium* all have a close evolutionary relationship with *Verrucomicrobium*.

### 3.3. Metagenome Function Prediction of the Degrading Bacteriophage TFM102

**Figure 7:**
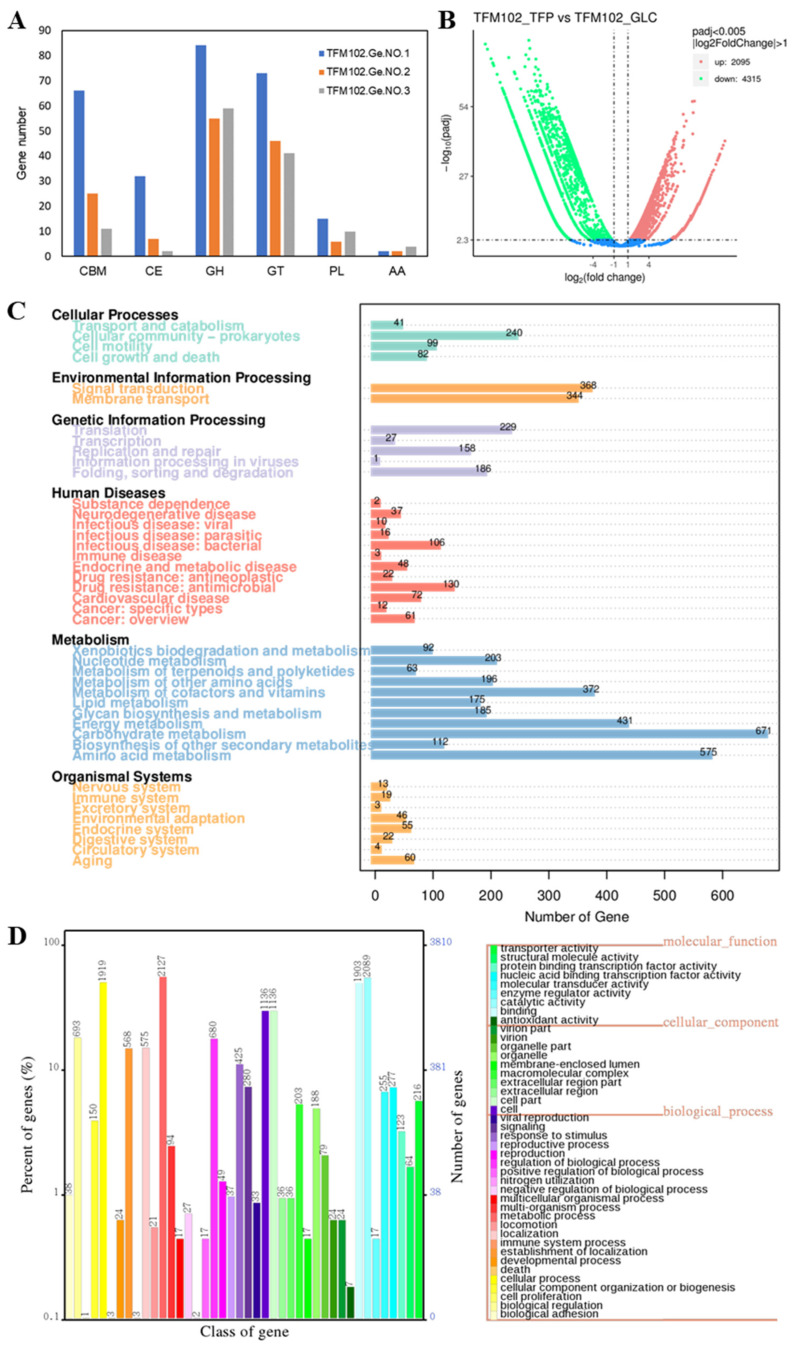
Gene function annotations by metagenome and macro-transcriptomics. (**A**) The bar chart illustrates the CAZy functional classification and the corresponding gene counts for each sample. (**B**) The volcano plot illustrates the overall distribution of genes with significant differential expression. Up-regulated genes are represented by red dots, while downregulated genes by green dots, and the blue dots represent non-differentiated genes. The statistical plot shows the number of genes annotated in each sample within the GO database (**C**) and the KEGG database (**D**). The numbers on the bars represent the counts of annotated genes.

Gene assembly of TFM102 was performed and three genomes were obtained, including *Verrucomicrobium* sp., *Lysobacter* sp., and *Sphingomonas* sp. It was consistent with the dominant species shown by microbiome analysis. According to the annotations of the CAZy database (Figure 7A), all three dominant bacteria contained abundant carbohydrate-active enzymes, especially GHs and glycosyltransferases (GTs). This means that polysaccharides are degraded to release sugar residues which bind to diverse substrates and participate in primary and secondary metabolic pathways. Among them, multiple carbohydrate-binding domains (CBMs) were also recognized in *Verrucomicrobium* sp., which are able to bind complex carbohydrates to enhance degradation. The degradation ability for the complex structure of TFPs by TFM102 may also benefit from the presence of CBMs.

The complex interlocking network in structural polysaccharides may resist chemical and enzymatic degradation and serve as a physical defense mechanism for the cell, which has been reported in algae [39]. It may explain the difficulty of degradation for complex polysaccharides in fungi. Complex carbohydrates consist of diverse monosaccharide subunits and glycosidic bonds that require a variety of specific enzymes for complete degradation and metabolism, and even demand different species to work at different steps for accomplishing synergistic utilization. Genes encoding carbohydrate-active enzymes are usually significantly enriched in saprophytic microorganisms degrading plant and fungal polysaccharides [40,41]. The annotation results of the metagenome showed that glycoside hydrolases are mainly concentrated in families GH 3, 5, 13, and 43. According to the CAZy database, families GH3 and GH5 cover rich species of glycoside hydrolases. Family GH5 is dominated by mannanase and glucanase, whereas family GH13 has multiple transferase functions and family GH43 is enriched with various exo-enzymes. Their functions were consistent with the structural characteristics of TFPs, which feature a mannan backbone enriched with a variety of side chains. The degradation of polysaccharides with multiple side chains and strong heterogeneity is a complex process, and the conversion of complex carbohydrates to monosaccharides usually requires the synergistic effects of multiple families.

Differential analysis and annotation of the macro-transcriptome demonstrated more functional information. Compared with the control sample cultured with glucose as the sole carbon source, 2095 genes were up-regulated after the induction of TFPs (Figure 7B). Based on the KEGG database, the biometabolism of TFM102 focused on carbohydrate (671), amino acid (575), and energy metabolism (431) (Figure 7C). GO annotations showed that the molecular functions of dominant binding (1903) and catalytic activity (2089) were prominent, implying active carbohydrate degradation (Figure 7D). According to the annotation of dbCAN, TFM102 was involved in the up-regulated expression of nearly thirty glycoside hydrolases from families GH16_3, GH47, and GH163 (Table S2) with activities of β-1,3/1,4-glucanase, α-1,2-mannosidase, and Endo-β-N-acetylglucosaminidase cleaving GlcNAc-β-1,2-Man. It has been demonstrated in prior research that the linkage types in TFPs include β-1,4-Glc*p* [42], α-1,2-man*p*, and a minor amount of acetylation [12]. The expression of over ten carbohydrate-binding modules from the families of CBM6, CBM9, and CBM16 was up-regulated. These proteins have the function of binding β-1,3/1,4-glucan, cellulose, and glucomannan, which can contribute to the efficient and stable degradation function of related hydrolases. In our previous work, it has been demonstrated that CBM enhance the binding of enzymes to their substrates, thereby increasing the catalytic efficiency and stability of the enzymes towards mannan and other complex polysaccharides [43,44]. Meanwhile, there was also a significant up-regulation of mannosyltransferases suspected to be from the GT32 and GT62 and the related genes, such as carbohydrate esterases (CEs), auxiliary activities (AAs), and polysaccharide Lyases (PLs). Multiple genes related to xylose and glucose metabolism were substantially up-regulated, as seen in the SwissProt annotation (Table S3), which suggested that the bacteria may have preferentially degraded the side chains of *Tremella fuciformis* mannans and mobilized the downstream metabolism of xylose and glucose residues. The up-regulation of the above functional genes suggested that addressing the challenges of controlled degradation and structural elucidation of TFPs requires the mining of diverse specific enzymes. The sequence of enzymatic degradation is also a critical aspect that deserves attention.

In conclusion, the complex action from multiple enzymes helped TFPs to be sufficiently degraded and converted into small molecule products to participate in bacterial metabolic pathways. In previous reports, the degradation of TFPs and the study of oligosaccharides were limited to their degradation methods. Although acid hydrolysis and oxidative degradation can effectively reduce the molecular weight of TFPs and produce small-molecule oligosaccharides, the degradation process and the oligosaccharide types are random and uncontrollable. It greatly increases the difficulty of structural analysis and limits the purification and application of oligosaccharides. The discovery of TFP-degrading bacteria and the analysis of their genomes provided the possibility of the enzymatically controllable degradation of TFPs.

## 4. Conclusions

In this study, we enriched and screened TF-degrading bacteria from soils in multiple locations, and successfully obtained a mixed bacterium. Its utilization for TFPs could reach more than 90% stably, and the extracellular enzyme released by it could realize the degradation of TFPs. The bacteria consisted of two dominant bacteria in similar proportions, *Verrucomicrobium* and *Lysobacter* with 43.8% and 55.4%, respectively. Active carbohydrate metabolism and prominent binding and catalytic functions were revealed using metagenomicsand macro-transcriptomics technologies combined with bioinformatics analysis. A variety of related degradative enzymes such as glucanase, xylanase, and mannanase were annotated, and their combined effects helped TFPs to be fully degraded and converted into small molecule products involved in microbial metabolism. This study obtained TFP-degrading bacteria for the first time, as well as revealed the mining potential of novel glycoside hydrolases among them, and recognized the potential proteins involved in the degradation process of TFPs. A foundation was created for the next step of mining TFP-degrading enzymes and broke through the bottleneck of enzymatic degradation of TF, which can be applied to TF viscosity reduction, oligosaccharide preparation, and structure elucidation. This provides more possibilities for the controlled degradation and application of TF macromolecular polysaccharides, and serves as a reference for the gene mining of glycoside hydrolases in microorganisms.

## Data Availability

The raw amplicon sequencing reads used in the manuscript are available at NCBI Sequence Read Archive under the BioProject accession number PRJNA1209679, and the raw metagenome sequencing under the BioProject accession number PRJNA1210843.

## Supplementary Materials

The following supporting information can be downloaded at: https://www.mdpi.com/article/10.3390/microorganisms13020263/s1, Table S1: Information on environmental microbial samples obtained from different regions and raw materials. Table S2: The dbCAN annotation of TFM102 differential genes. Table S3: The SwissProt annotation of carbohydrate-related enzymes in the TFM102 differential gene.
