# Supplementary material for: Microbiota Involved in the Degradation of Tremella fuciformis Polysaccharide and Microbial Enzymatic Potential Revealed by Microbiome and Metagenome
Source: Microorganisms. 2025 Jan 25;13(2):263. doi: 10.3390/microorganisms13020263 (PMC11858309; doi:10.3390/microorganisms13020263)
Supplement: Supplementary file 1 [file microorganisms-13-00263-s001.zip › microorganisms-3429776-supplementary.pdf]

Supplementary Table

Table S1. Information on environmental microbial samples obtained from different regions and raw materials.

simple number	source	material
1	Ningde Fujian	Substitute material
2	Ningde Fujian	Substitute material
3	Guangzhou Guandong	Substitute material
4	Linyi Shandong	Substitute material
5	Linyi Shandong	Substitute material
6	Laoshan	Soil
7	Laoshan	Soil
8	Xiaozhushan	Soil
9	Taishan	Soil
10	Jiuzhaigou	Soil

Table S2. The dbCAN annotation of TFM102 differential genes.

Gene ID	log2FoldChange	dbCAN
TFM102.Ge.NO.1_GM004156	9.90	CBM16
TFM102.Ge.NO.1_GM001908	6.99	GT60
TFM102.Ge.NO.1_GM003928	6.67	GH163
TFM102.Ge.NO.1_GM006707	6.34	CBM6
TFM102.Ge.NO.1_GM006448	6.20	GH50
TFM102.Ge.NO.1_GM001701	6.16	CBM9
TFM102.Ge.NO.1_GM001736	5.91	GH171
TFM102.Ge.NO.1_GM004119	5.79	GH16_3
TFM102.Ge.NO.1_GM004301	5.66	GH110
TFM102.Ge.NO.2_GM000501	5.48	GH47
TFM102.Ge.NO.1_GM003842	5.44	PL8_3
TFM102.Ge.NO.1_GM003196	5.36	CBM51
TFM102.Ge.NO.1_GM000507	5.12	GH165
TFM102.Ge.NO.1_GM003034	5.05	CBM47
TFM102.Ge.NO.1_GM003919	5.03	GH94
TFM102.Ge.NO.1_GM005204	4.98	GT32, GT62
TFM102.Ge.NO.1_GM005560	4.89	GH33
TFM102.Ge.NO.1_GM004111	4.47	GH33
TFM102.Ge.NO.1_GM000310	4.45	GT51
TFM102.Ge.NO.1_GM006792	4.28	GT51
TFM102.Ge.NO.1_GM006490	4.20	GH165
TFM102.Ge.NO.1_GM001646	4.13	CBM47, CBM47
TFM102.Ge.NO.1_GM005636	4.06	GH77
TFM102.Ge.NO.1_GM000754	4.03	GH33
TFM102.Ge.NO.1_GM004032	3.86	PL9_1
TFM102.Ge.NO.2_GM000742	3.85	GH3
TFM102.Ge.NO.1_GM000556	3.66	PL38
TFM102.Ge.NO.1_GM003859	3.52	PL8_2
TFM102.Ge.NO.1_GM002830	3.49	GT2
TFM102.Ge.NO.1_GM003801	3.32	GT4
TFM102.Ge.NO.1_GM006210	3.28	PL15
TFM102.Ge.NO.1_GM006631	3.20	CBM67
TFM102.Ge.NO.1_GM004286	3.18	GH33
TFM102.Ge.NO.1_GM001575	3.12	GT83
TFM102.Ge.NO.1_GM006050	3.02	GT51
TFM102.Ge.NO.1_GM003643	2.89	GH13_3
TFM102.Ge.NO.1_GM005579	2.77	GH16_3
TFM102.Ge.NO.1_GM000976	2.63	PL6_1
TFM102.Ge.NO.1_GM006891	2.61	GH33, GH165
TFM102.Ge.NO.1_GM005541	2.60	GH33, CE6
TFM102.Ge.NO.2_GM001882	2.60	GT84, GH94
TFM102.Ge.NO.1_GM003835	2.48	GH50
TFM102.Ge.NO.1_GM004860	2.48	PL1_2
TFM102.Ge.NO.1_GM001615	2.44	GT2, GT2
TFM102.Ge.NO.1_GM003868	2.42	PL15
TFM102.Ge.NO.2_GM001182	2.38	GT41

TFM102.Ge.NO.2_GM002375	2.34	GT51
TFM102.Ge.NO.2_GM002797	2.29	GH23
TFM102.Ge.NO.1_GM004411	2.24	CBM32
TFM102.Ge.NO.1_GM005591	2.23	GH18
TFM102.Ge.NO.1_GM003998	2.17	GH5
TFM102.Ge.NO.1_GM000946	2.16	GT35
TFM102.Ge.NO.1_GM001903	2.08	CBM38
TFM102.Ge.NO.1_GM006596	2.05	GT9
TFM102.Ge.NO.1_GM002181	1.93	CBM32
TFM102.Ge.NO.1_GM002839	1.91	GH163
TFM102.Ge.NO.1_GM002459	1.85	GH33
TFM102.Ge.NO.1_GM002893	1.81	GT83
TFM102.Ge.NO.2_GM000592	1.66	PL1_2
TFM102.Ge.NO.1_GM004110	1.63	GH33
TFM102.Ge.NO.1_GM005039	1.61	CBM47
TFM102.Ge.NO.1_GM006089	1.37	CBM38
TFM102.Ge.NO.1_GM001710	1.21	PL10_1
TFM102.Ge.NO.2_GM002531	1.13	GH23
TFM102.Ge.NO.2_GM001487	1.02	GH31

Table S3. The swissprot annotation of carbohydrate-related enzymes in the TFM102 differential gene.

gene ID	log2FoldChange	swissprot
TFM102.Ge.NO.1_GM005956	6.34	Xylose isomerase (EC 5.3.1.5)
TFM102.Ge.NO.1_GM006037	5.86	Acetylxylan esterase (EC 3.1.1.72) (Acetyl-xylooligosaccharide esterase)
TFM102.Ge.NO.1_GM004140	5.55	D-xylonolactone lactonase (EC 3.1.1.110) (Xylono-1,5-lactonase),6-deoxy-6-sulfogluconolactonase (EC 3.1.1.99) (6-deoxy-6-sulfogluconolactone lactonase) (SGL lactonase)
TFM102.Ge.NO.1_GM002786	4.39	Xylulose kinase (Xylulokinase) (EC 2.7.1.17)
TFM102.Ge.NO.1_GM000141	3.93	Xyloglucanase (EC 3.2.1.-)
TFM102.Ge.NO.1_GM001776	3.66	Phosphoglucomutase (PGM) (EC 5.4.2.2) (Alpha-phosphoglucomutase) (Glucose phosphomutase)
TFM102.Ge.NO.1_GM005269	3.63	6-phosphogluconolactonase (EC 3.1.1.31)
TFM102.Ge.NO.1_GM004069	3.55	1,5-anhydro-D-fructose reductase (Anhydrofructose reductase) (EC 1.1.1.292) (1,5-anhydro-D-fructose reductase (1,5-anhydro-D-mannitol-forming))
TFM102.Ge.NO.1_GM000923	3.40	6-deoxy-6-sulfo-D-gluconate dehydratase (SG dehydratase) (EC 4.2.1.162)
TFM102.Ge.NO.1_GM005442	3.18	Arabinose-proton symporter (Arabinose transporter)
TFM102.Ge.NO.1_GM002291	3.11	Sulfatase (EC 3.1.6.-) (Arylsulfatase) (Polysaccharide utilization locus H protein P14) (PUL H protein P14) (Sulfatase family S1 subfamily 7 protein P14) (P14_S1_7)
TFM102.Ge.NO.1_GM000372	2.84	Glucose-6-phosphate isomerase (GPI) (EC 5.3.1.9) (Phosphoglucose isomerase) (PGI) (Phosphohexose isomerase) (PHI), Glucose-6-phosphate isomerase (GPI) (EC 5.3.1.9) (Phosphoglucose isomerase) (PGI) (Phosphohexose isomerase) (PHI), Glucose-6-phosphate isomerase (GPI) (EC 5.3.1.9) (Phosphoglucose isomerase) (PGI) (Phosphohexose isomerase) (PHI)
TFM102.Ge.NO.2_GM001148	2.77	Glucose transport protein

TFM102.Ge.NO.1_GM005228	2.62	N-acetylgalactosamine-6-sulfatase (EC 3.1.6.4) (Chondroitinsulfatase) (Chondroitinase) (Galactose-6-sulfate sulfatase) (N-acetylgalactosamine-6-sulfate sulfatase) (GalNAc6S sulfatase), N-acetylgalactosamine-6-sulfatase (EC 3.1.6.4) (Chondroitinsulfatase) (Chondroitinase) (Galactose-6-sulfate sulfatase) (N-acetylgalactosamine-6-sulfate sulfatase) (GalNAc6S sulfatase)
TFM102.Ge.NO.2_GM000142	2.54	Phosphogluconate dehydratase (EC 4.2.1.12)
TFM102.Ge.NO.2_GM000144	2.46	Glucose-6-phosphate 1-dehydrogenase (G6PD) (EC 1.1.1.49)
TFM102.Ge.NO.1_GM002506	2.45	Phosphorylated carbohydrates phosphatase TM_1254 (EC 3.1.3.-)
TFM102.Ge.NO.1_GM004613	2.45	GDP-L-fucose synthase 1 (EC 1.1.1.271) (GDP-4-keto-6-deoxy-D-mannose-3,5-epimerase-4-reductase 1) (AtFX) (AtGER1)
TFM102.Ge.NO.1_GM001450	2.41	Carbonic anhydrase 2 (EC 4.2.1.1) (Carbonate dehydratase 2)
TFM102.Ge.NO.1_GM003885	2.32	Beta-1,4-N-acetylgalactosaminyltransferase 3 (B4GalNAcT3) (Beta4GalNAc-T3) (Beta4GalNAcT3) (EC 2.4.1.244) (Beta-1,4-N-acetylgalactosaminyltransferase III) (N-acetyl-beta-glucosaminyl-glycoprotein 4-beta-N-acetylgalactosaminyltransferase 2) (NGalNAc-T2)
TFM102.Ge.NO.1_GM001816	1.71	Glucans biosynthesis glucosyltransferase H (EC 2.4.1.-)
TFM102.Ge.NO.1_GM001089	1.61	N-acetylglucosamine-6-O-sulfatase (EC 3.1.6.-)
TFM102.Ge.NO.1_GM004896	1.52	Bifunctional sulfatase/alpha-L-rhamnosidase (P36_GH78/S1_25) [Includes: Ulvan-active sulfatase (EC 3.1.6.-) (Sulfatase family S1 subfamily 25 protein P36) (P36_S1_25); Alpha-L-rhamnosidase (EC 3.2.1.40) (Glycosyl hydrolase 78 family protein P36) (P36_GH78) (Polysaccharide utilization locus H protein P36) (PUL H protein P36)]
TFM102.Ge.NO.1_GM000498	1.34	Quinoprotein glucose dehydrogenase (EC 1.1.5.2) (Glucose dehydrogenase [pyrroloquinoline-quinone])

TFM102.Ge.NO.1_GM005249	1.33	Probable sugar efflux transporter,Probable sugar efflux transporter
